# Barriers and facilitators of implementing public–private mix approaches for active tuberculosis case finding and health insurance access in at-risk populations in Ghana: a qualitative study

**DOI:** 10.3389/frhs.2025.1738753

**Published:** 2026-01-12

**Authors:** Kenneth Mawuta Hayibor, Ernest Kenu, Delia Akosua Bandoh, Benedicta Owusu-Arthur, Magdalene Akos Odikro, Gloria Ivy Mensah, Dziedzorm Awalime, Adwoa Asante-Poku, Olena Ivanova, Andrea Rachow, Nii Nortey Hanson-Nortey

**Affiliations:** 1 Center for International Health, Ludwig-Maximilians-Universität, Munich, Germany; 2 Noguchi Memorial Institute for Medical Research, University of Ghana, Accra, Ghana; 3 School of Public Health, University of Ghana, Accra, Ghana; 4 Aurum Institute, Accra, Ghana; 5 Institute of Infectious Diseases and Tropical Medicine, LMU University Hospital, LMU Munich, Munich, Germany; 6 German Centre for Infection Research (DZIF), Partner Site Munich, Munich, Germany; 7 Unit of Global Health, Helmholtz Zentrum München, German Research Centre for Environmental Health (HMGU), Neuherberg, Germany

**Keywords:** active case-finding, community-based screening, facilitators and barriers, health insurance, public-private mix, tuberculosis

## Abstract

**Background:**

In Ghana, although free tuberculosis (TB) services are provided at public facilities, the TB case detection rate is still lower than anticipated. To enhance TB case detection, private community healthcare providers and the National Health Insurance Scheme (NHIS) have been involved using a Public-Private Mix (PPM) model, which includes active case finding (ACF) and access to insurance. This study examines the facilitators and barriers to implementing a PPM model that aims to expand ACF and provide health insurance to newly diagnosed TB patients among at-risk populations in two Ghanaian cities.

**Methods:**

This was an exploratory qualitative study based on 54 TB patient exit interviews, key informant interviews from seven sub-metro TB coordinators and 44 facility-level TB coordinators, and six focus group discussions were held, comprising four with health workers (*n* = 53) and two with volunteers (*n* = 18). We conducted a thematic content analysis and, based on the key themes identified, we applied the Consolidated Framework for Implementation Research (CFIR) to structure the themes across five domains.

**Results:**

Implementation of the PPM model was facilitated by strong stakeholder collaboration, adaptable screening procedures, adequate diagnostic resources, and effective supervision. However, delays in NHIS reimbursements, limited registration logistics, weak intersectoral communication, and high staff turnover constrained implementation. While community engagement and the integration of TB screening into routine care enhanced uptake, persistent financial and operational barriers limited the program's sustainability.

**Conclusion:**

The PPM model increased TB case detection and expanded insurance coverage but was limited by structural inefficiencies, especially within NHIS operations. Future efforts should address systemic misalignments, support healthcare workers, and improve NHIS-private provider collaboration. Recognizing facilitators and barriers can help policymakers, TB program managers, NHIS officials, and partners plan more effective PPM models to boost active case finding in Ghana and similar settings.

## 1 Introduction

Tuberculosis (TB) continues to pose a significant public health challenge, particularly in low- and middle-income countries (LMICs), where at-risk populations face substantial barriers to diagnosis, treatment, and care (1). Despite global progress in reducing TB incidence and mortality, gaps in case detection and healthcare coverage persist, limiting the effectiveness of TB control programs (2, 3).

Active case finding (ACF) has been identified as a vital strategy to bridge these gaps by identifying individuals with TB who may not actively seek care (4, 5). However, the effectiveness of ACF depends heavily on the extent to which programs can overcome system-level barriers and ensure the meaningful engagement of diverse stakeholders (6–9). Many ACF strategies in LMICs lack coordinated engagement with private sector providers, who are often the first point of contact for individuals with presumptive TB (10). This gap highlights the need for innovative models that integrate public and private actors to strengthen TB detection and linkage to care.

To effectively engage stakeholders, Public-private mix (PPM) models have become an effective ACF approach to strengthen TB control efforts (11, 12). Existing evidence demonstrates that PPM models enhance TB detection and treatment by leveraging private sector capacity, resulting in higher referrals, improved adherence to national guidelines, and stronger surveillance (13, 14). However, the effectiveness of PPM models varies greatly across different settings, mainly due to context-specific implementation challenges, including misaligned incentives, weak policy support, inconsistent provider engagement, and patient-level barriers such as cost and trust (15). While several studies have evaluated outcomes, such as TB notifications under PPM (10, 16, 17), less is understood about how and why these models succeed or fail during implementation, especially in the context of ACF.

In Ghana, efforts to enhance TB control include integrating private healthcare providers and implementing financial protection mechanisms through the National Health Insurance Scheme (NHIS) (18). Introduced in 2003 as part of the country's progress toward universal health coverage, NHIS coverage remains suboptimal, with enrollment lowest among the urban poor populations that are simultaneously at higher risk of TB (19). To address these gaps, a targeted PPM model was implemented in two cities, involving private healthcare providers who screened clients and community members for TB, with NHIS reimbursing consultation and case-review costs for enrolled patients. This model was designed not only to enhance ACF but also to reduce financial barriers to care.

This study aims to examine the facilitators and barriers to implementing a PPM model designed to scale up ACF and increase NHIS enrollment among at-risk populations in two cities in Ghana. Utilizing qualitative methods, we explore the perspectives of patients, private healthcare workers, and program coordinators to understand how implementation processes, contextual factors, and stakeholder experiences influence the success of the model. By identifying critical determinants of implementation, this study contributes new evidence to inform the design, scale-up, and sustainability of the PPM model for TB control in Ghana and other LMIC settings.

## 2 Study methods

### 2.1 Study design

This qualitative descriptive study sought to explore the implementation of the PPM model and the provision of health insurance for tuberculosis care. The study design allowed for an in-depth understanding of the facilitators and barriers encountered by three stakeholder groups: tuberculosis patients, health workers/volunteers, and sub-metro TB coordinators. Data were collected through semi-structured interviews and focus group discussions (FGDs). By triangulating information across the different stakeholder groups, the validity of the findings was enhanced. The analysis followed the guidelines of the Consolidated Framework for Implementation Research (CFIR), which facilitated the interpretation of the findings within the relevant implementation contexts. The CFIR is a conceptual framework developed to guide the systematic assessment of different implementation contexts to identify key factors that may influence the implementation and effectiveness of a given intervention (20).

### 2.2 Setting of the study and intervention pathway

The PPM model was implemented in five sub-metropolitan (sub-metros) assemblies in Accra and four in Kumasi, the two major cities in Ghana from October 2018 to March 2020. These specific sub-metros have been selected based on the features of high-burden TB communities and low case detection rates. Despite having all the features of high-burden TB settings, the selected intervention metros in Accra and Kumasi carry 14% of the national population but reported only 9.8% of TB notification in 2017.

**Figure 1:**
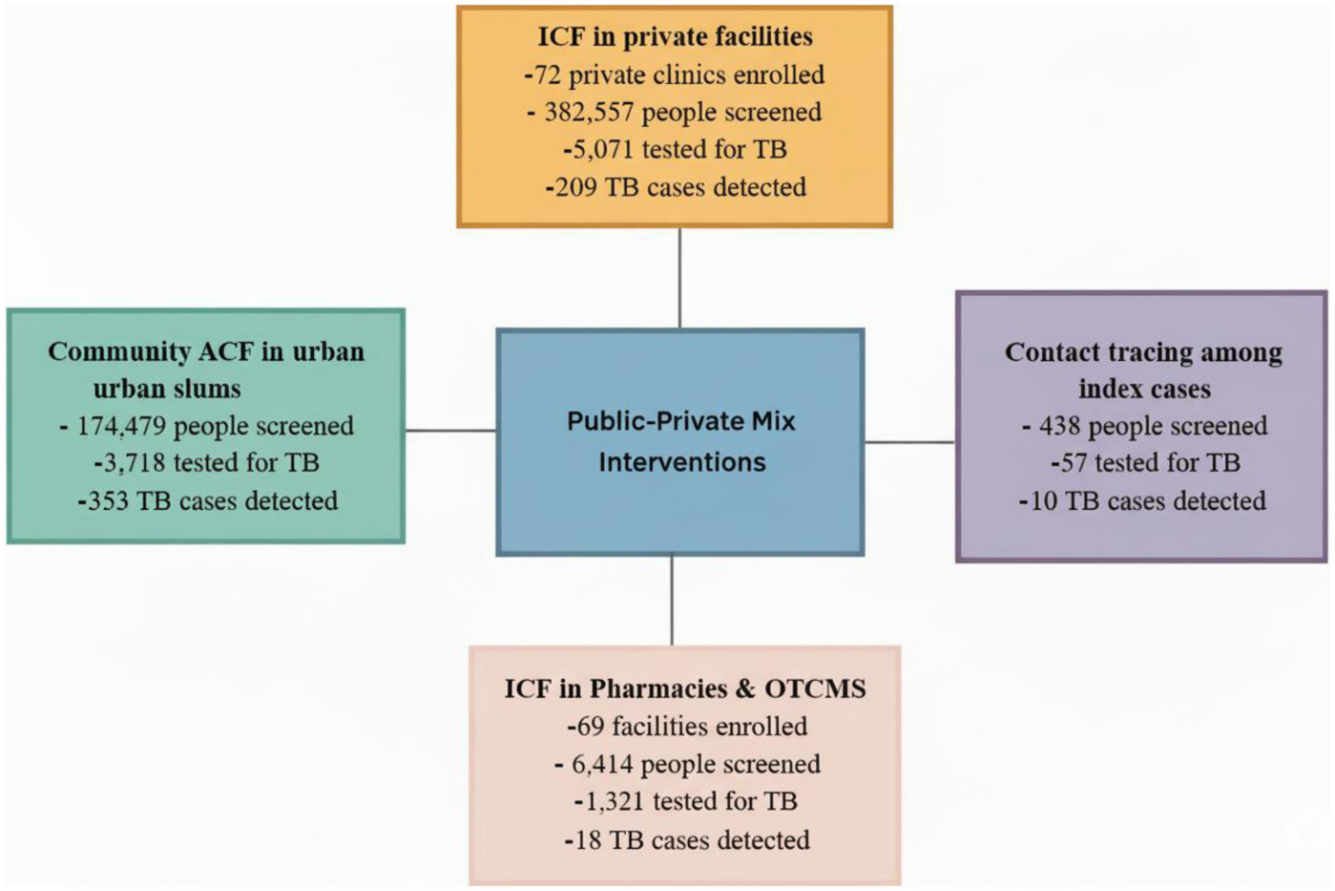
The private-public mix model interventions implemented with screening outcomes.

The PPM model intervention was implemented in 72 private health facilities, 69 pharmacies and Over-The-Counter Medicines Shops (OTCMS), and slum communities by the Aurum Institute Ghana (AIG) in collaboration with two local NGO partners – Ghana TB Voice Network (TBVN) and Afro Global Alliance (AGA). Key interventions involving public and private healthcare providers areas focused on: (1) intensified case finding in private health facilities, (2) intensified case finding in community pharmacies and over-the-counter medicine sellers (OTCMS), (3) community active case search in urban slums, and (4) contact tracing among index TB cases. The details, yield, and impact of each intervention have been previously described (21); however, we provide a brief overview of the interventions’ screening outcomes in Figure 1.

Health workers in private facilities were trained and supported to screen for TB at their Outpatient Departments (OPDs). Through the launch of “Know Your Lung” campaigns in all the sub-metros, trained community volunteers increased TB awareness and generated traffic to accredited private health clinics and community pharmacies as entry points for TB screening and case finding. Presumed patients were screened, tested, and those diagnosed with TB were enrolled in free TB treatment and NHIS to cover review consultations in private clinics. The community volunteers were also assigned to clinics, pharmacies, and the communities to refer presumptive TB cases for testing, transport samples, conduct home visits, and contact investigations.

### 2.3 Participants selection and recruitment

Participants were purposively sampled to reflect the perspectives of three key stakeholder groups: (1) TB patients identified through the PPM intervention, (2) health workers and community volunteers engaged in the PPM interventions, and (3) sub-metro and facility-level TB coordinators in the PPM intervention areas.

Patients were recruited if they were currently on TB treatment or had recently completed treatment at one of the participating private healthcare facilities. Eligibility was verified using TB treatment cards and patient folders. Health workers and volunteers were selected from facilities and communities actively involved in screening, referral, and treatment activities. Implementers, comprising all sub-metro TB coordinators and facility-level TB coordinators in the intervention sites, were also approached to participate, given their direct involvement in project oversight and delivery.

### 2.4 Data collection

Data collection took place from October 2019 to November 2022 at intervention facilities and communities involved. Semi-structured interview guides with open-ended questions were developed for each stakeholder group through a review of relevant literature on PPM and ACF implementation, informed by the study's conceptual framework and implementation outcomes of interest, and further refined through expert input. The guides were piloted and adjusted before data collection. The patient interview guide explored experiences with TB screening, diagnosis, treatment, and NHIS enrolment. The interview guides for health workers, volunteers, and coordinators explored various factors that influenced the implementation of the interventions and their outcomes, including intervention characteristics, acceptability, adoption, processes, sustainability, successes, challenges, and areas for improvement. Interview guides were translated into local languages (Twi and Ga) and back-translated into English to ensure accuracy and cultural relevance. To strengthen trustworthiness, triangulation was applied by drawing information from different stakeholder groups and validating patient inclusion through treatment cards and facility records.

A team of four qualified research assistants with at least a bachelor's or master's degree in public health were recruited. They received intensive training in qualitative research methods, interviewing techniques, and ethical conduct during a two-day preparatory workshop. Mock interviews were conducted during training to standardize approaches. Research assistants explained the study purpose to participants before interviews, emphasizing that the aim was to capture their lived experiences rather than test their knowledge.

All interviews and FGDs were conducted face-to-face in private spaces such as health facility offices, pharmacies, or homes, based on convenience and confidentiality. No invited participants declined; some requested rescheduling due to other commitments, which were accommodated. Interviews lasted 15–30 min, FGDs lasted 60–90 min.

All interviews were audio-recorded with the participants’ consent and were supplemented by field notes taken immediately afterward. These field notes included contextual information and observations that were important for data interpretation. Data collection was conducted iteratively, with interviews and FGDs continuing until no new information or themes emerged, confirming saturation. In total, we conducted 54 exit interviews with TB patients and key informant interviews with seven sub-metro TB coordinators and 44 facility-level TB coordinators. Additionally, we organized four FGDs with health workers (*n* = 53) and two with volunteers (*n* = 18), taking place in both Accra and Kumasi to capture shared experiences and group dynamics. The overview of the study participants interviewed is presented in Table 1.

**Table 1:** Overview and characteristics of interviewees.

Stakeholder group	Number	Number and proportion of female participants
Patients	54	20 (34.5%)
Health workers and volunteers		
Health workers	53	39 (73.3%)
Volunteers	18	8 (44.4)
Coordinators		
Facility	44	29 (65.9)
Sub-metro	7	2 (28.6)
Total	176	98 (55.7)

This exploratory approach allowed for diverse perspectives to be documented, ensuring a deeper understanding of both patient experiences and implementation dynamics within the PPM model.

### 2.5 Data analysis

The interviews and discussions were audio-recorded and transcribed verbatim by the research assistants and imported into NVivo qualitative data analysis software. Field notes were taken after every FGD and used in the analysis. To ensure anonymity and confidentiality, interviewees were coded by as numbers, and identifiers were removed. Thematic content data analysis of the transcripts was conducted by the any two of the authors HKM, DAB, BOA, MAO and cross-checked by EK to resolve any difference. To ensure all themes were identified, we used an inductive approach for thematic analysis. The authors discussed the final themes and categorized them into main themes and sub-themes based on their relevance to the research question posed by the study.

Following a thematic content analysis based on the final themes reported by participants in this study, we identified the CFIR as the most suitable model to provide a theoretical basis for interpreting the findings (21). The CFIR was chosen because it provides a comprehensive, multi-domain framework that is especially useful for evaluating complex public–private implementation models like the PPM intervention. Its ability to incorporate multiple stakeholder perspectives and systematically identify barriers and facilitators at the intervention, organizational, and individual levels made it the most suitable framework for understanding the implementation dynamics observed in this study. The CFIR consists of five major domains that may affect implementation including: (1) intervention characteristics; (2) outer setting; (3) inner setting; (4) characteristics of individuals; and (5) the implementation process. Definitions of the five domains and their subconstructs are further detailed in Supplementary Table S1. Within each domain, we assessed key factors or constructs of the intervention that influence implementation.

### 2.6 Reflexivity statement

The research team acknowledges that our backgrounds and training may have influenced the study's design, data collection, and interpretation. The lead author is a public health researcher with experience in implementing health interventions. While this expertise enhanced the analysis, it also may pose a risk of bias. To mitigate this, five of the authors, who are mainly from diverse disciplines, participated in coding and validating findings. We held regular team discussions to ensure interpretations reflected participants’ perspectives, and field research assistants, not involved in TB service delivery, conducted most interviews to reduce social desirability bias.

### 2.7 Ethics

Written informed consent was obtained from all study participants. Ethical clearance was obtained from the GHS Ethics Review Committee (GHS-ERC 00s/01/19) and an ethical vote from Ludwig-Maximilians-University Munich, Germany (23–0484). The interventions and operational research were funded by the TB REACH initiative of the Stop TB Partnership Wave 6. The sponsor had no role in the study design or data collection. The sponsor was involved in monitoring overall project performance through quarterly reports but was not involved in analysis, decision to publish, or preparation of the manuscript.

## 3 Results

The implementation of the PPM model intervention to scale up active TB case finding and to provide health insurance coverage for TB patients revealed both interrelated facilitators and barriers. These factors span individual behaviors, community dynamics, and organizational structures. A thematic analysis identified a combination of these facilitators and barriers that influenced implementation across various levels of the health system. The findings were organized according to the five key domains and 16 constructs of the CFIR, which highlighted the intricate interplay among intervention characteristics, contextual influences, and the implementation processes, categorizing them as either barriers or facilitators (Table 2). In the following section, we summarize the findings according to each domain of the CFIR framework. The first domain outlines the characteristics of the PPM model, while the subsequent domains detail the specific facilitators and barriers encountered.

**Table 2:** Factors influencing the implementation of the public-private mix model for active TB case detection and health insurance access.

CFIR domain	Facilitators	Barriers
Intervention characteristics		
Origin	The interventions were developed by the lead implementers (Aurum Ghana), the NTP, NHIS and all relevant stakeholders	
Adaptability	Screening process and procedure, and NHIS enrollment, were adapted to suit health facilities and enhance client retention.	
Relative advantage	Implementing facilities were able to drive demand for their services within their communities. Patients enrolled with NHIS can regularly seek free healthcare at any implementing facility Utilizing TB screening to educate patients and community members on other conditions.	.
Outer setting		
Critical incidents		Delay in the transportation of samples. Delay in claims processing and payments
Partnerships and collaborations	Good network and referral between stakeholders	Difficulty in getting NHIS officials to register patients
Financing	Timely disbursement of funding from the lead implementers	Delay in reimbursement of claims from the NHIS
Inner setting		
Available resources	Provision of screening materials and diagnostic GeneXpert machines	Limited availability of NHIS registration kits at private healthcare facilities.
Access to Knowledge & Information	The capacity of healthcare staff and volunteers to screen for TB, fill NHIS claim forms was enhanced, but	There was a high turnover of healthcare staff.
Communications	The implementation facilitators regularly visited sites, but Volunteers were from the same communities, they spoke mainly local dialects, and used their networks in recruiting participants	Poor coordination between NHIS and private healthcare providers led to confusion in processing claims. There were challenges in communicating major changes to private healthcare providers.
Incentive Systems	Private healthcare providers were incentivized	Health workers were not adequately compensated for additional work, even though their institutions were compensated
Individuals		
Implementation coordinators	Field officers, project managers, and TB coordinators were highly engaged in supervising screening activities and addressing operational challenges.	NHIS officers were not consistently available to support health insurance registration, causing delays in patient enrollment.
Intervention deliverers	Private healthcare providers and community health volunteers were instrumental in conducting TB screening, but high staff turnover affected continuity.	NHIS officials had difficulty coordinating with private healthcare providers, leading to delays in processing claims and new registrations.
Intervention recipients	Willingness of some community members to undergo TB screening due to awareness campaigns and perceived health benefits.	Stigma surrounding TB prevented some individuals from accessing screening services. Many vulnerable populations lacked awareness of NHIS registration processes, leading to lower uptake of insurance. Financial constraints still limited some individuals from seeking care despite NHIS coverage due to transportation and informal charges.
Implementation process		
Teaming	All private healthcare providers registered with their professional association were invited to willing participate in the TB case finding. Collaboration with community-based organizations helped to increase awareness and patient referrals.	
Assessing needs	The community intervention did not align with a need expressed by the community.	Many individuals perceived NHIS registration as needless, as they will still pay out of pocket for health services
Planning and engaging	All stakeholders were engaged in the operational planning to implement TB case detection. All stakeholders were encouraged to register with the NHIS to accept patients with insurance Engaging municipal health authorities helped strengthen support for private sector providers.	Hesitancy of some private healthcare providers to engage the NHIS due to past negative experiences with delayed reimbursements

### 3.1 Innovation characteristics

The successful implementation of the innovation (PPM model intervention) can be attributed to its key characteristics and the collaborative design that involved multiple stakeholders. Within the innovation characteristics domain, three constructs: origin, adaptability, and relative advantage, were particularly critical to successful implementation. The specific characteristics of the PPM model related to these constructs are discussed below.

#### 3.1.1 Origin

Interviewees indicated that the PPM model, which provides free TB services, enrolls diagnosed patients, and offers health insurance via the NHIS, is a well-designed intervention led by AIG in partnership with civil society organizations, the NTP, and the NHIS. They believe it should be supported and expanded to different regions across the country.

“Right from the beginning… we were involved in designing the interventions. Our inputs were factored in…sometimes we attended meetings with them while our personnel from the NTP national office were also present. “ (TB Coordinator, KII)

The incorporation of NHIS enrollment for TB patients was established in partnership with NHIS by AIG, aiming to provide free healthcare access to at-risk patients, particularly those with TB, by reimbursing consultation costs.

“NHIS officials were involved in the planning, I know they all signed the MoU (memorandum of understanding), NHIS, as we know was to pay for the consultation fees of the TB patients” (TB Coordinator, KII)

#### 3.1.2 Adaptability

The screening tool and process were adapted to suit the operations of private healthcare facilities, allowing for flexibility in how TB screening was conducted. This adaptability was a key factor in increasing participation from private providers.

“We were able to adjust the screening to fit into our workflow, sometimes during vitals, other times during consultation, depending on how busy we were. The tool made it easy to adapt as needed.” (Healthworker, FGD)

#### 3.1.3 Relative advantage

Private healthcare providers noted that implementing TB screening increased their visibility and increased demand for their services within their communities. Additionally, screening sessions became an opportunity to educate patients about other health conditions. For NHIS enrollment, the main advantage was that patients with insurance could regularly seek free healthcare, reducing financial barriers to treatment adherence.

“Once people realized we offered TB screening, they started coming in not just for that but for other health issues as well.” (Healthworker, FGD)

“Now that the hospital (private facility) accepts NHIS, I can go to the clinic without worrying about money. Before, I would have avoided that hospital.” (TB Patient, KI)

### 3.2 Outer setting

The implementation of the PPM model for TB case detection and health insurance access was significantly influenced by external contextual factors. Participants described a combination of facilitators and barriers that affected collaboration, funding, and service delivery within the broader health system.

#### 3.2.1 Outer setting facilitators

Several external factors facilitated successful implementation. The most notable was the strong collaboration and partnerships between stakeholders**,** including the lead implementers (AIG), private healthcare providers, and NTP. These relationships strengthened referral systems, improved coordination of TB case detection, and fostered trust among partners.

“I think with the commencement of the program, there has been a massive improvement with regard to detection and treatment. The private facilities worked well with each other (Sub-metro TB coordinator KII)”

The timely disbursement of funds and provision of diagnostic resources by the lead implementers were also key facilitators. The timely disbursement ensured that screening materials were always available, allowing for uninterrupted TB testing and diagnosis.

“AURUM provided money for the supply of materials needed for TB testing in our facility. This has helped in making the diagnosis faster (Health worker, FGD)

Additionally, regular follow-up visits, coordination meetings, and feedback sessions between the implementers, NTP, and private providers ensured that screening guidelines and operational plans were well understood. These interactions encouraged active participation and improved the responsiveness of the system.

#### 3.2.1 Outer setting barriers

Challenges within the outer setting created barriers to effective implementation. The most significant was the impact of the COVID-19 pandemic, which severely disrupted community outreach, sample transportation, and NHIS registration activities. Lockdowns and staff shortages reduced access to services and delayed TB diagnosis.

“During the lockdowns, everything slowed down. We couldn't go out to screen people, and even when we did and send the samples to the lab, getting results became a challenge.” (Volunteer, FGD)

“COVID made everything difficult. Patients couldn't even register for NHIS properly because offices were closed or had reduced staff.” (Healthworker, FGD)

Another major barrier was weak collaboration with NHIS offices, which led to persistent delays in claim reimbursements and patient registration. These administrative bottlenecks discouraged some private healthcare providers from actively participating in the program.

“I sent the list there, but my clients have not yet been enrolled; the process delayed a lot.” (Sub-metro Coordinator, KII)

Financial and logistical challenges further constrained implementation. Although Aurum Ghana provided funds for TB activities, the NHIS reimbursement process was slow, and registration materials such as forms and kits were often unavailable in private facilities. This hindered efforts to enroll patients on-site and undermined the integration of health insurance into TB care.

“We wanted to register them in our facility, but the NHIS officials were not around, and we do not have the registration kits like the big hospitals.” (Nurse, FGD)

### 3.3 Inner setting

Participants emphasized that effective implementation of the interventions relied on the availability of resources, accessible knowledge and information, and clear communication. Conversely, they noted barriers such as inadequate incentives for workers despite a heavier workload, delayed communication from lead implementers to volunteers, and negative attitudes from some volunteers.

#### 3.3.1 Inner setting facilitators

The availability of resources has proven to be a key factor in enhancing TB screening and diagnosis. The introduction of GeneXpert machines, screening tools, and the transportation of samples from facilities without GeneXpert to those equipped with it by the lead implementers significantly bolstered the capacity of facilities to detect TB. Participants noted that these resources not only improved the accuracy and speed of diagnosis but also encouraged private providers to stay involved in the intervention.

“The Aurum people (lead implementers) have provided 4 GeneXpert machines, which have helped in the diagnosis. We suggested that since not all facilities have the machines, they should introduce sample transportation from the facilities without the machines to the facilities with the machines. Of course, we recommended the staff be remunerated as this is additional workload” (TB coordinator, KII)

Access to knowledge and training was another major facilitator. Health workers and volunteers received orientation on TB screening protocols, data recording, and NHIS claim documentation. This enhanced their confidence and skills in implementing the intervention.

“The Aurum staff trained us on how to use the screening tools and complete the claim forms.” (Health Worker, FGD)

Effective communication among stakeholders significantly enhanced implementation. Participants highlighted that regular supervision and feedback from AIG and the NTP provided clarity on program expectations, which in turn maintained motivation among providers.

“The Aurum people have maintained good communication with all stakeholders right from the beginning of the intervention. They always asked for our inputs and feedback as the implementation was going on.” (Sub-metro TB Coordinator, KII)

#### 3.3.1 Inner setting barriers

Several barriers within the inner setting limited the effective implementation. These included high staff turnover, poor communication between institutions, inadequate incentives, and logistical gaps in NHIS registration.

High turnover of healthcare staff in private facilities posed a serious challenge. Frequent staff departures created disruptions in service continuity and required repeated retraining, which slowed down implementation.

“Our biggest issue is retaining staff; we spend time training them and the Aurum staff also trained them on TB. Some of our staff left to government hospitals and some even travelled out of the country. We had to keep training new staff, and that slowed things down in the facility alongside the TB screening.” (Submetro coordinator-KII)

Another key barrier was inadequate incentives for frontline health workers. Although private facilities received institutional compensation, many staff members felt that their individual contributions were not fairly recognized or rewarded, despite the increased workload of screening and documentation.

“We have added the TB screening to our work. It is not only about asking the basic screening questions or showing the TB screening diagrams, for each patient we have to fill the TB register documenting each patient. We are doing more work, but we are not seeing any extra pay. It's frustrating.” (Nurse, FGD)

Logistical barriers hindered the integration of NHIS in private facilities. Some providers lacked registration kits and forms, while NHIS officials were often unavailable to assist with on-site enrollment.

“We are doing the screening and so far have recorded some cases, but the NHIS officials were not around, and we don't have registration kits like the big hospitals.” (Nurse, FGD)

### 3.4 Individuals

The individuals involved in implementing the PPM model, including project coordinators, TB officers, private healthcare providers, community volunteers, NHIS officers, and patients, played a crucial role in shaping how the intervention was delivered. Their motivation, engagement, and capacity either facilitated or constrained the effectiveness of the model.

#### 3.4.1 Individual domain facilitators

A key facilitator was the strong commitment and engagement of field officers, project managers, and TB coordinators who provided supervision, mentoring, and technical support throughout the intervention. Their active involvement ensured that operational challenges were promptly addressed and that screening processes remained consistent across facilities.

“The TB team was always there to help; they supervised, supported us, and made sure we followed the right procedures.” (TB Coordinator, KII)

Private healthcare providers and community health volunteers also played important facilitator roles. Their close connection to community members and familiarity with local contexts enhanced trust and increased participation in TB screening and NHIS registration. Volunteers’ ability to communicate in local dialects helped reduce fear and improve patient follow-up.

“Because I am from this community, I know everyone here. We work as a team and help each other follow up with clients. There is a good relationship and network among us.” (Volunteer, FGD)

Additionally, awareness campaigns and the perceived health benefits motivated many community members to undergo TB screening. Patients who had been educated about TB symptoms or had witnessed successful treatment outcomes were more willing to participate and enroll in NHIS.

“When they explained about TB and the test was free, I decided to do it. It's better to know than to keep coughing and not be sure.” (TB patient, KII)

#### 3.4.2 Individual domain barriers

A major barrier was the inconsistent availability of NHIS officers. Their irregular presence in facilities caused delays in patient registration and discouraged some providers from integrating NHIS enrollment into routine workflows.

“The TB team was always there to help, but NHIS? Sometimes you couldn't even find them when you needed them.” — (TB Coordinator, KII)

High turnover of private healthcare staff and volunteers also affected program continuity. The frequent replacement of trained personnel disrupted screening consistency and increased the need for repeated retraining, which slowed progress.

“There's no motivation. This is extra work for the facility, and many people resign, so new ones have to be trained again.” (Health Worker, FGD)

Among patients, stigma, misinformation, and financial barriers were key obstacles. Fear of discrimination prevented some individuals from participating in TB screening, while limited awareness of NHIS registration processes led to low enrollment. Even insured patients still faced indirect costs such as transportation and informal charges, which discouraged service utilization.

“People are scared of being labeled as having TB, so they avoid testing.” (Volunteer, FGD)

“Even with NHIS, I still need money for transport and other charges. It's not completely free.” (TB Patient, KII)

### 3.5 Implementation process

The implementation process captures how the PPM intervention was planned, executed, and evaluated across private healthcare facilities and communities. Participants described several processes that facilitated effective implementation, as well as barriers that limited the smooth rollout and sustainability of the intervention.

#### 3.5.1 Implementation process facilitators

The inclusive planning and engagement of stakeholders emerged as a central facilitator in the implementation process. TB coordinators, private healthcare providers, and NHIS representatives were involved in operational planning and decision-making from the early stages. This participatory approach promoted shared ownership, improved coordination, and fostered commitment to the success of the program.

“We were involved in the decisions, particular on how to increase the number of people we screen and how to better engage the zongo communities” (TB coordinator, KII)

Integration of TB screening and NHIS registration into routine facility activities was another key facilitator. Health workers reported that TB screening had been smoothly incorporated into daily outpatient services, making the intervention sustainable within existing workflows.

“Basically, we do the screening as we were trained by Aurum. It starts from the OPD, and from there, the necessary questions are asked. Integrating this into our activities is smooth, although it's a lot of work.” (Health Worker, FGD)

Furthermore, strong teamwork and coordination among private providers enhanced program delivery. Providers collaborated within their professional networks to share resources, maintain consistency, and enhance service quality.

#### 3.5.2 Barriers

Hesitancy among some private healthcare providers to engage with the NHIS due to previous negative experiences with delayed claim reimbursements. This mistrust reduced enthusiasm for participation and slowed NHIS integration was a major barrier.

“I was hesitant to accept with the introduction of NHIS to cover TB patients also due to my negative experience with long delays in NHIS reimbursement.” (Health Worker, FGD)

Limited alignment of the intervention with perceived community needs also emerged as a barrier. Some community members did not view TB screening or NHIS registration as a priority, especially when they believed healthcare costs would still be paid out-of-pocket.

“NHIS doesn't feel useful to me, people still end up paying for many things out of pocket, so it's not a priority.” (TB Patient, KII)

Bureaucratic and logistical constraints in linking NHIS services to private facilities further delayed implementation. Participants emphasized the need for NHIS representation within facilities to allow real-time registration and insurance renewal.

“The process for NHIS registration and renewal needs to be carried out at the facilities to ensure it is fast and convenient for the patients.” (Health Worker, FGD)

## 4 Discussion

This qualitative study explored stakeholders’ perceptions of the facilitators and barriers to implementing a PPM model designed to scale up ACF and provide health insurance (NHIS) coverage for newly diagnosed vulnerable TB patients in Ghana. Using the CFIR, we found that while stakeholders broadly recognized the PPM model as innovative and ensured collaboration to easily screen patients/clients for TB, its implementation revealed significant complexity due to contextual, structural, and individual-level factors.

Overall, our research supports the findings of earlier studies in Ghana regarding the facilitators and barriers to ACF in healthcare facilities (22–24). Similarly, some of the findings are consistent with studies conducted in other countries (25–31), as well as the evidence presented in systematic and scoping reviews (32, 33). In studies conducted in Ghana and other parts of Africa, the identified facilitators include effective communication and referral systems, health workers’ awareness of the need for interventions, and the involvement of chemical sellers. Others outside Africa include the use of digital referral and notification platforms, which reduce delays in diagnosing presumptive TB cases. Conversely, the barriers recognized involve the absence of medical insurance, perceived stigma, insufficient TB diagnostic resources in rural health facilities, a disorganized standard referral system, inadequate training in case detection guidelines, and fear of infection. As these factors were known before the initiation of the ACF interventions in our study, we leveraged the facilitators. We aimed to address the barriers by introducing health insurance for newly diagnosed TB patients, providing regular training and updates on TB screening tools, conducting community programs to educate community members about TB, supplying new diagnostic machines, and implementing a sample transportation system.

The study findings indicated that implementing an ACF through the PPM model and integrating health insurance relies heavily on stakeholder engagement to meet the set targets. A significant contributor was the flexible nature of TB screening processes, which enabled private healthcare providers to seamlessly incorporate them into their routine activities with minimal disruption. The design of the interventions included input from various stakeholders, promoting strong ownership and commitment. Previous studies have shown similar results, wherein participatory planning enhanced the uptake and sustainability of interventions (28, 30, 34). Additionally, engaging the community through private healthcare providers increased the visibility and credibility of these facilities, which in turn improved patient attendance and expanded their role in TB detection. However, adapting NHIS processes to fit the workflows of private facilities faced challenges. Providers encountered bureaucratic delays, ambiguous reimbursement mechanisms, and inconsistent communication, all of which diminished trust and decreased their willingness to fully engage with the NHIS component. These challenges align with prior findings in health systems research, where integrating insurance schemes into private healthcare delivery has often encountered operational hurdles (39).

Expanding access to healthcare services and resources is crucial for addressing the needs of vulnerable populations. Strategies such as enhancing TB diagnostic tools and algorithms, along with involving all care providers, are recommended to identify missing TB cases (35). Resource availability is vital for the success of any TB-ACF intervention, regardless of the model used (36). In our study, we observed that quickly delivering samples to laboratories equipped with GeneXpert machines allowed for accurate diagnoses in private settings. In community outreach programs and other private facilities lacking GeneXpert, sample transportation was established to ensure timely diagnoses without adding burden to patients or clients. A significant resource challenge was the absence of NHIS registration kits and personnel in nearly all private health facilities, which impeded real-time patient enrollment and reinforced the perception that the insurance system remains inaccessible. Lack of medical insurance limits efforts to combat TB. Uninsured individuals often travel further to public providers, whereas insured individuals have access to a wider range of providers, including private ones, and may travel less, reducing transportation costs (37).

The study found that personal attitudes and community views greatly influenced implementation outcomes of the PPM model. Health workers and volunteers were motivated to implement the interventions, yet they highlighted the absence of incentives despite increased workloads. Furthermore, despite initial training for private healthcare workers, high turnover rates compromised continuity and quality, underscoring the need for continuous capacity building, especially in the facilities, where staff retention frequently poses a challenge. Although patients and community members were open to TB screening thanks to awareness campaigns, they still had concerns about residual costs and the stigma associated with TB. These results align with existing literature emphasizing the significance of perceived value and trust in health interventions (38).

It has become increasingly evident in our study that to effectively scale up the PPM model and enhance active TB case finding and health insurance coverage for at-risk populations, sustained and deliberate actions are essential, not just the identification of facilitators but overcoming the barriers. Achieving impactful implementation outcomes requires a co-creation approach that fully engages stakeholders, including private healthcare providers, NHIS officials, TB program managers, and community representatives, in collaboratively tackling challenges and aligning on common goals. For example, in the initial stages of implementing the interventions, we identified the issue of losing presumptive patients who need to submit samples to facilities with the GeneXpert machine, as well as delays in sample submissions that resulted in delayed diagnoses. We resolved this issue by collaborating with all facilities, both public and private, to introduce sample transport and expedite turnaround times for results. A major challenge that the interviewees consistently highlighted as a significant barrier to success, which still needs to be fully addressed, is the difficulty in enrolling TB clients onto the NHIS platform. Although the aim to combine TB services with insurance coverage is commendable, issues such as poor follow-through, bureaucratic inefficiencies, and insufficient on-site registration support have significantly diminished its potential effectiveness.

To move forward, it is essential to establish functional, decentralized NHIS registration systems within private facilities, with clearly defined roles for NHIS officers and digital tools to streamline the process. Regular joint review meetings among stakeholders can also help track progress, adjust strategies, and foster collective accountability. Additionally, targeted incentives to reach the health workers and volunteers, task-shifting, and enhanced data-sharing mechanisms between private facilities and national programs could help build trust and increase participation in both TB screening and NHIS registration. Integrating such solutions into implementation plans will not only strengthen the PPM model but also cultivate local ownership and sustainability of interventions.

### 4.1 Strengths and limitations

This study adds value by presenting specific insights into identifying barriers and leveraging facilitators while suggesting ways to address barriers. A key strength lies in involving various stakeholder groups, including community volunteers, who are engaged in the PPM model throughout its design and implementation. The four multifaceted interventions proved easily adaptable by private healthcare providers. Capacity-building initiatives and access to diagnostic tools, such as GeneXpert machines and screening instruments, enabled early detection of TB cases. Collaboration with skilled data collectors, familiar with qualitative research, contributed to the production of high-quality, contextualized data for our study. Data triangulation and thorough discussions among research team members helped us gain a clearer understanding of the reliability, accuracy, and comprehensiveness of the data collected.

In terms of study limitations, it is worth noting that the capacity building, public engagement, and diagnostic components of the PPM model were externally funded, which affects its scalability and long-term sustainability. Mobilizing national resources to support all aspects of the PPM model interventions would be advantageous. Another limitation was the time gap between the project's conclusion and the study's duration, potentially introducing recall bias. To reduce this risk, interviewers employed probing techniques and cross-verified responses among participant groups to improve accuracy and ensure that accounts were as detailed and consistent as possible. Although issues with enrolling patients into the NHIS were a major barrier, we were unable to interview any NHIS personnel to understand this setback.

## 5 Conclusions

This study shows that although the PPM model improved TB screening and fostered better collaboration among stakeholders, its overall success was limited by ongoing structural, financial, and operational challenges, especially those related to NHIS processes, which restricted its full potential. Achieving integrated care, including the effective use of NHIS for TB services, will require addressing these systemic misalignments and improving coordination between public and private sector actors. Moving forward, strengthening structural support, communication pathways, and financial systems will be essential to sustain and scale PPM interventions. These findings offer actionable guidance for policymakers, national TB program managers, NHIS authorities, and implementing partners seeking to strengthen structural support, communication pathways, and financial systems to sustain and scale PPM-based ACF interventions in Ghana and similar low- and middle-income country settings.

## Data Availability

The raw data supporting the conclusions of this article will be made available by the authors, without undue reservation.
