# Supplementary material for: Barriers and facilitators of implementing public–private mix approaches for active tuberculosis case finding and health insurance access in at-risk populations in Ghana: a qualitative study
Source: Front Health Serv. 2026 Jan 12;5:1738753. doi: 10.3389/frhs.2025.1738753 (PMC12832754; doi:10.3389/frhs.2025.1738753)
Supplement: Supplementary file 1 [file Table1.docx]

Table 1: Consolidated Framework for Implementation Research domain description

| **Domain/Construct Name** | **Construct Definition (***The degree to which:)* | |
| --- | --- | --- |
| **I. INNOVATION DOMAIN (*Innovation:*** The “thing” being implemented) | | |
| A. Innovation Source | | The group that developed and/or visibly sponsored use of the innovation is reputable, credible, and/or trustable. |
| C. Innovation Relative Advantage | | The innovation is better than other available innovations or current practice. |
| D. Innovation Adaptability | | The innovation can be modified, tailored, or refined to fit local context or needs. |
| **II. OUTER SETTING DOMAIN (*Outer Setting:*** The setting in which the Inner Setting exist) | | |
| A. Critical Incidents | | Large-scale and/or unanticipated events disrupt implementation and/or delivery of the innovation. |
| D. Partnerships & Connections | | The Inner Setting is networked with external entities, including referral networks, academic affiliations, and professional organization networks. |
| F. Financing | | Funding from external entities (e.g., grants, reimbursement) is available to implement and/or deliver the innovation. |
| **III. INNER SETTING DOMAIN (*Inner Setting:*** The setting in which the innovation is implemented) | | |
| C. Communications | | There are high quality formal and informal information sharing practices within and across Inner Setting boundaries (e.g., structural, professional). |
| H. Incentive Systems | | Tangible and/or intangible incentives and rewards and/or disincentives and punishments support implementation and delivery of the innovation. |
| J. Available Resources | | Resources are available to implement and deliver the innovation. |
| K. Access to Knowledge & Information | | Guidance and/or training is accessible to implement and deliver the innovation. |
| **IV. INDIVIDUALS DOMAIN (*Individuals:*** The roles and characteristics of individuals) | | |
| D. Implementation Facilitators | | Individuals with subject matter expertise who assist, coach, or support implementation. |
| H. Innovation Deliverers | | Individuals who are directly or indirectly delivering the innovation. |
| I. Innovation Recipients | | Individuals who are directly or indirectly receiving the innovation. |
| **V. IMPLEMENTATION PROCESS DOMAIN (*Implementation Process:*** The activities and strategies used to implement the innovation) | | |
| A. Teaming | | Join together, intentionally coordinating and collaborating on interdependent tasks, to implement the innovation. |
| B. Assessing Needs | | Collect information about priorities, preferences, and needs of people. |
| D. Planning | | Identify roles and responsibilities, outline specific steps and milestones, and define goals and measures for implementation success in advance. |
| F. Engaging | | Attract and encourage participation in implementation and/or the innovation. |
